# 2361. Prevalence of Zoster Infection Post mRNA COVID-19 Vaccination Compared to Influenza Vaccination

**DOI:** 10.1093/ofid/ofad500.1982

**Published:** 2023-11-27

**Authors:** Mark S Lazbin, David C Kaelber

**Affiliations:** MetroHealth Medical Center, Copley, Ohio; MetroHealth Medical Center/ Case Western Reserve University, Cleveland, Ohio

## Abstract

**Background:**

**Introduction:** Varicella-zoster virus (VZV) reactivation was not listed as a side effect of any vaccine until introduction of COVID-19 vaccines. Since COVID-19 vaccines were introduced, several case reports described the link between COVID-19 vaccination and VZV reactivation. In our study we aimed to investigate how often patients developed VZV reactivation after mRNA COVID-19 vaccine compared to influenza vaccine.

**Methods:**

We used the TriNetX platform and its global health research network containing aggregated de-identified information of electronic health records on over 100 million patients from scores of healthcare organizations. Three patient groups were identified:

Patients receiving their 1^st^ mRNA COVID-19 vaccine 12/20/2020-12/20/2022

Patients receiving their 2^nd^ mRNA COVID-19 vaccine 12/20/2020-12/20/2022

Patients receiving influenza vaccine 12/20/2018-12/20/2022

Propensity Score Matching was used for age, gender, and Zoster vaccine status.

We examined the prevalence of VZV reactivation in each group during the first 21 days following vaccination.

**Results:**

Out of 444,016 patients in each matched group receiving either their 1^st^ mRNA COVID-19 vaccine or the Influenza vaccine, 0.04% in the COVID-19 vaccine group and 0.15% in the influenza group, odds ratio (OR) 0.24 (95% CI 0.20-0.29), were diagnosed with VZV reactivation within 21 days. Among 445,382 patients in each matched group receiving either their 2^nd^ mRNA COVID-19 vaccine or their influenza vaccine, 0.04% in the COVID-19 vaccine group and 0.13% in the Influenza group, OR 0.27 (95% CI 0.22-0.32), were diagnosed with VZV reactivation within 21 days. There was a lower risk of VZV reactivation after 1^st^ or 2^nd^ dose of mRNA COVID-19 vaccine than influenza vaccine.

**Conclusion:**

The risk of VZV reactivation after the 1^st^ or 2^nd^ dose of mRNA COVID-19 vaccination is smaller than VZX reactivation after influenza vaccine. The risk of VZV reactivation after mRNA COVID-19 vaccine is very low and hesitance to receive COVID-19 vaccine due to concern of VZV reactivation is not substantiated.

**Disclosures:**

**David C. Kaelber, MD, PhD, MPH, FAAP, FACP, FACMI, FAMIA**, Becton, Dickinson and Company: Advisor/Consultant|Dynavax Technologies Corporation: Advisor/Consultant|Merck Sharp & Dohme Corporation: Advisor/Consultant

